# Removal of a Pessary after Forty-one Years’ Residence in the Pelvis

**Published:** 1852-09

**Authors:** 


### OBSTETRICS.

*Removal of a Pessary after forty-one years' residence in the Pelvis.*—In the *Ohio Medical and Surgical Journal* for May, Dr. F. T. Hurxthal reports the case of a German woman, aged 73 years, who, in 1811, after a confinement, had prolapsus uteri; and upon consulting a midwife (in Germany) she introduced a ring into the vagina, which she said was made of wood covered with beeswax. It had never been removed, nor had she ever felt the least inconvenience from it, until, on the second day of an attack of pneumonia, whilst coughing violently, she felt some pain in the pelvic region, which increasing, was followed by an exceedingly fetid vaginal discharge. The pessary was removed with difficulty, by division of it by scissors. It was 3⅝ inches in its diameter, and 2⅞ in its transverse diameter. On removal, it presented the appearance of a roughened stone, in some parts over an inch in thickness. The recovery was speedy and complete.

